# Clindamycin plus quinine for treating uncomplicated falciparum malaria: a systematic review and meta-analysis

**DOI:** 10.1186/1475-2875-11-2

**Published:** 2012-01-04

**Authors:** Charles O Obonyo, Elizabeth A Juma

**Affiliations:** 1 Centre for Global Health Research, Kenya Medical Research Institute, P.O. BOX 1578, Kisumu 40100, Kenya; 2 Division of Malaria Control, P.O. BOX 19982, Nairobi 00202, Kenya

## Abstract

**Background:**

Artemisinin-based combinations are recommended for treatment of uncomplicated falciparum malaria, but are costly and in limited supply. Clindamycin plus quinine is an alternative non-artemisinin-based combination recommended by World Health Organization. The efficacy and safety of clindamycin plus quinine is not known. This systematic review aims to assess the efficacy of clindamycin plus quinine versus other anti-malarial drugs in the treatment of uncomplicated falciparum malaria.

**Methods:**

All randomized controlled trials comparing clindamycin plus quinine with other anti-malarial drugs in treating uncomplicated malaria were included in this systematic review. Databases searched included: Cochrane Central Register of Controlled Trials, MEDLINE, EMBASE and LILACS. Two authors independently assessed study eligibility, extracted data and assessed methodological quality. The primary outcome measure was treatment failure by day 28. Dichotomous data was compared using risk ratio (RR), in a fixed effects model.

**Results:**

Seven trials with 929 participants were included. Clindamycin plus quinine significantly reduced the risk of day 28 treatment failure compared with quinine (RR 0.14 [95% CI 0.07 to 0.29]), quinine plus sulphadoxine-pyrimethamine (RR 0.17 [95% CI 0.06 to 0.44]), amodiaquine (RR 0.11 [95% CI 0.04 to 0.27]), or chloroquine (RR 0.11 [95% CI 0.04 to 0.29]), but had similar efficacy compared with quinine plus tetracycline (RR 0.33 [95% CI 0.01 to 8.04]), quinine plus doxycycline (RR 1.00 [95% CI 0.21 to 4.66]), artesunate plus clindamycin (RR 0.57 [95% CI 0.26 to 1.24]), or chloroquine plus clindamycin (RR 0.38 [95% CI 0.13 to 1.10]). Adverse events were similar across treatment groups but were poorly reported.

**Conclusion:**

The evidence on the efficacy of clindamycin plus quinine as an alternative treatment for uncomplicated malaria is inconclusive. Adequately powered trials are urgently required to compare this combination with artemisinin-based combinations.

## Background

Malaria is a disease of global public health importance, caused by protozoan parasites of the genus *Plasmodium*. The epidemiology of malaria is rapidly changing and elimination may be feasible in some endemic regions within the next decade. Over 90% of the malaria burden is borne by populations in sub-Saharan Africa where *Plasmodium falciparum *is predominant and the high risk groups include young children and pregnant women [1]. In the absence of a vaccine, chemotherapy plus vector control remain the main tools for reducing malaria-related morbidity and mortality in Africa. Some studies linked the emergence of anti-malarial (specifically, chloroquine) drug resistance to an increased incidence of severe malarial anaemia and malaria-related mortality [2-5].

Artemisinin-based combination therapy (ACT) is the recommended standard of care in the treatment of uncomplicated falciparum malaria [6,7]. The adoption of combination therapy -the simultaneous administration of two or more blood schizontocidal drugs with independent modes of action and different biochemical targets in the parasite - is thought to improve treatment efficacy and to delay the emergence of drug resistance to the individual components of the combination [8,9]. However, the implementation of ACT policy in the African public health sector is challenged by limited availability (resulting in frequent stock-outs), inaccessibility and high cost of the drugs [10]. The situation may be improving due to increased financial and technical support from global initiatives, such as the Global Fund for AIDS, TB and Malaria, and the Affordable Medicines for Malaria. In some settings, the effective implementation of ACT for malaria treatment and insecticide-treated bed nets for vector control has already resulted in substantial reductions of malaria-related morbidity, mortality and admissions [11-14]. ACT resistance has been described and global efforts are underway to contain the evolution of artemisinin resistance [15,16]. Alternative combinations to ACT are necessary and should be assessed for efficacy and safety. Clindamycin plus quinine is a potential non-ACT combination recommended by World Health Organization (WHO)[7].

Clindamycin is a lincosamide antibiotic with anti-malarial activities. It is used for the treatment of anaerobic and gram positive bacterial infections, toxoplasmosis, babesiosis, and *Pneumocystis carinii *pneumonia. The drug is available in formulations suitable for oral (as capsules or oral suspension), or for parenteral administration. Clindamycin is effective against *P. falciparum*, even as monotherapy, but it is a slow-acting drug with a mean parasite clearance time of four to six days and a mean fever clearance time of three to five days. When used as monotherapy, it must be given twice daily for at least 5 days. In general, clindamycin is a well-tolerated drug with mild and transient side effects. Initial studies described an association linking clindamycin therapy with a diarrhoeal illness due to *Clostridium difficile*, but this is rare and the most frequent side effects include anorexia, nausea, vomiting and abdominal discomfort [17].

Quinine is a fast-acting drug with a short elimination half-life that has been recommended for the treatment of severe malaria (in all age groups), for uncomplicated malaria in pregnant women and for drug-resistant malaria for almost 400 years [18]. Quinine is available as an oral, rectal or parenteral formulation and is administered eight hourly per day for 7 days [19]. The most frequent adverse event associated with quinine treatment is cinchonism, which is characterized by tinnitus, nausea, headache and blurred vision. Combinations of quinine with some antibiotics (e.g., tetracycline, doxycyline, clindamycin and azithromycin) significantly improved the treatment efficacy compared to quinine alone in the treatment of drug-resistant malaria [20-22]. The continued use of quinine is however challenged by poor tolerability and poor compliance associated with the duration of treatment and the complex dosing regimes [23,24].

Clindamycin plus quinine qualifies as an anti-malarial drug combination because both drugs have potent anti-malarial properties but different mechanisms of action and the relatively fast-action of quinine can overcome the drawback arising from the slow-action of clindamycin. In combination with clindamycin, the treatment course for quinine can be shortened to 3 days, which may improve adherence, making this combination a more practical and efficient option to consider for the treatment of uncomplicated malaria [25]. In addition, this combination can be administered to both children and pregnant women (in all the three trimesters).

However, it is unclear whether this combination can be a safe and effective alternative to ACT. In the absence of evidence from a systematic review, the malaria treatment guidelines by WHO have recommended clindamycin plus quinine as the drug of choice for treatment of malaria infection in the first trimester of pregnancy and as a second-line anti-malarial drug for other cases, based on consensus opinion [7]. The objective of this systematic review was to assess the available evidence on the efficacy and safety of clindamycin plus quinine compared to other anti-malarial drugs (alone or in combination) when used for treating adults and children with uncomplicated falciparum.

## Methods

### Search strategy and selection criteria

Studies were considered for inclusion in the review if they were randomized controlled trials designed to compare the efficacy of clindamycin plus quinine with another anti-malarial drug (used alone or in combination) in participants with symptomatic, microscopically-confirmed uncomplicated falciparum malaria. Studies that had enrolled participants with signs of severe malaria were excluded. The review's primary outcome was prospectively defined as parasitological treatment failure evaluated 28 days after starting treatment. Secondary outcomes included day 14 parasitological failure, gametocyte carriage, parasite and fever clearance time, mean haemoglobin and adverse events.

Using a combination of the terms, "malaria"," quinine", "clindamycin" or" dalacin", a search of the following electronic databases was made: Cochrane Infectious Diseases Group specialized register; Cochrane Central Register of Controlled Trials (CENTRAL), published in *The Cochrane Library 2011, Issue 3*; MEDLINE (1966 to October 2011); EMBASE (1988 to October 2011); and LILACS (1982 to October 2011). The third, fourth and fifth MIM Pan-African Malaria Conference proceedings were also searched for relevant abstracts. Individual researchers working in the field, organizations including WHO and the East African Network for Monitoring Antimalarial Treatment (EANMAT), and pharmaceutical companies including Pfizer, Sanofi-Aventis, Novartis, and Rhône-Poulenc Rorer were contacted for unpublished and ongoing trials. The reference lists of retrieved studies were also checked for additional studies. An attempt was made to retrieve all relevant trials regardless of language or publication status (published, unpublished, in press, and in progress).

### Data extraction and management

Abstracts obtained by the search strategy were screened for potentially relevant trials. Full text articles of the selected trial reports were retrieved. Each trial report was scrutinized for multiple publications from the same data set. If a trial was published more than once, only one publication was assessed. Based on the inclusion criteria, both authors independently assessed the trials for inclusion in the review. For the included trials, data on trial dates, location, publication status, trial methods, participants, interventions and outcomes was independently extracted by both authors onto data abstraction forms. Any disagreements were resolved by referring to the study report and by discussion. Additional information was sought from the trial authors if the available data was insufficient or missing.

Data was extracted to allow for an intention-to-treat analysis. For dichotomous outcome, the number of participants experiencing the event and the number analysed in each treatment group was recorded. For continuous outcome measures (e.g. fever and parasite clearance times), the arithmetic means and standard deviations for each group was extracted in addition to the numbers analysed in each group. The range was extracted if medians were reported.

### Assessment of the methodological quality of included studies

The methodological quality for each included trial was independently assessed by both authors. Specifically, the following components of methodological quality were assessed: generation of allocation sequence, allocation concealment, blinding and loss to follow up. Generation of allocation sequence and allocation concealment were classified as adequate, inadequate, or unclear [26]. Blinding was classified as open, single, or double blind. The percentage of randomized participants lost to follow up was calculated, and the percentage of participants available for analysis was categorized as adequate, if it was at least 90%.

### Data analysis

Data was analysed using Review Manager 5.1 software. Dichotomous data was combined using risk ratios (RR), while continuous data was combined using the weighted mean difference (WMD). Where arithmetic means were reported for an outcome measure where the scale is naturally bound at zero, the ratio of the mean to the standard deviation was used to check the assumption that the data are normally distributed. When data was suspected to be skewed or inappropriately summarized as means and standard deviations (mean/sd > 2) then it was not combined in a meta-analysis. The results are presented as point estimates together with the 95% confidence intervals. P values less than 0.05 were considered statistically significant differences. A fixed effects model was used in pooling data where there was no evidence of heterogeneity.

Statistical heterogeneity was assessed by visually inspecting the forest plots (for overlapping confidence intervals), applying the chi-square test (p value < 0.10 considered statistically significant), and the *I*^2 ^statistic with the value of 50% used to denote moderate levels of heterogeneity [27]. If heterogeneity was detected, and if it was still appropriate to pool the data, then a random-effects model was used [28]. Potential sources of heterogeneity were explored by conducting subgroup analyses using participant age (less than versus greater or equal to 5 years), and the effect of different drug dosing regimes on treatment efficacy. A sensitivity analysis was performed using allocation concealment to evaluate the robustness of our conclusions.

## Results

### Description of included studies

Seventeen potentially relevant titles were found. Five articles were immediately excluded at the initial screening stage because they did not meet the inclusion criteria. Four other trials were excluded because they had no comparison groups [29-32], and one because it had assessed only safety outcomes and did not report efficacy data [33].

This review is based on the seven randomized trials that met the study inclusion criteria. The eligible trials had enrolled 929 participants and had been conducted between 1987 and 2004 in Gabon [34-36], Thailand [37,38], France [39] and Brazil [40]. Of the 929 participants randomized in these trials, 826 (88.9%) were included in the analysis, indicating an overall attrition rate of 11% (range 1.5 to 21%).

All the included trials had enrolled participants with microscopically-confirmed *P. falciparum *infection. Adult participants were studied in all the trials, except for two trials from Gabon, where children were enrolled [34,36]. Of the five trials with adult participants, one had enrolled travellers returning from the tropics [39], while the other had enrolled pregnant women [38]. The trials varied in their definition for when adult age began: 14 years in one trial [40], 15 years in two trials [35,39] and unspecified in two trials [37,38]. The ages of the enrolled children ranged from three to 12 years in one trial [36] and four to 15 years in another [34]. The sample size for the included trials was generally small, ranging from 100 to 204 participants.

In three trials, the study design included three parallel treatment arms in which clindamycin plus quinine was compared with: quinine alone or quinine plus doxycycline [35], quinine alone or quinine plus tetracycline [37]; amodiaquine alone or quinine plus sulphadoxine-pyrimethamine [40]. In two trials, clindamycin plus quinine was compared with artesunate alone [38], or clindamycin plus artesunate [36]. In one trial [39], intravenous quinine plus intravenous clindamycin was compared with intravenous quinine followed by oral quinine. The last trial had four treatment arms, in which clindamycin plus quinine was compared with quinine alone, chloroquine alone, or a combination of chloroquine plus clindamycin [34].

In four trials, clindamycin plus quinine was administered every 12 h for 3 days [34-36,40]. Clindamycin plus quinine was administered every 8 hours for 3 days in one trial [39], and for seven days in another trial [38]. In the last trial, clindamycin was given every six hours, while quinine was administered every 8 hours, for 7 days [37]. Overall, treatment duration ranged from 3 to 7 days, and the total daily dose for clindamycin ranged from 10 to 30 mg/kg per day.

The duration of follow up was 28 days after initiation of treatment in six trials [34-37,39,40] and 42 days in one trial [38]. The primary endpoint was defined as day 28 parasitological failure in five trials [34,35,37,39,40], day 28 parasitological cure (polymerase chain reaction [PCR]-corrected) in one trial [36] and day 42 parasitological failure (PCR-corrected) in one trial [38]. All the seven trials reported on parasite clearance time and adverse events, three trials reported on fever clearance time [36,37,39], two trials on day 14 parasitological failure [34,35], two trials on the mean haemoglobin [36,38], one trial on progression to severe anaemia [38], and two trials on gametocyte carriage [36,38]. However, parasite clearance time (PCT) and fever clearance time (FCT) were not always consistently reported. Some trials reported the mean PCT [35,36,38,40], while others reported the median value [34,39]. Only one trial reported the mean FCT [36].

Only two trials performed an intention-to-treat analysis [36,39]. The remaining five trials reported outcomes only for evaluable participants. Three trials revealed their source of funding, including two that had received funding from the Wellcome Trust of Great Britain [37,38], and one, that had been funded by the local government in France [39]. Table 1 is a summary of the characteristics of the included studies.

**Table 1:** Characteristics of the included trials

Reference	Location/dates	Participants	Interventions	Outcomes
40	Brazil (1987)	Adults (> 14 yrs)	Quinine +clindamycin [1] Amodiaquine (AQ) Quinine + SP	Day 28 parasite failure, PCT, adverse events

34	Gabon (1992)	Children (4-15 yrs)	Quinine+clindamycin [2] Quinine Chloroquine (CQ) CQ + clindamycin	Day 28 parasite failure, Day 14 parasite failure, PCT, adverse events

35	Gabon (1993-1994)	Adults (> 15 yrs)	Quinine + clindamycin [2] Quinine Quinine + doxycycline (Dx)	Day 28 parasite failure, Day 14 parasitological failure, PCT, adverse events

37	Thailand (1995-1997)	Adult males	Quinine +clindamycin [3] Quinine Quinine + tetracycline (Tx)	Day 28 parasite failure, PCT, FCT, adverse events

39	France (1996-1998)	Returning travelers	Quinine +clindamycin [4] Quinine	Day 28 parasite failure, PCT, FCT, adverse events

38	Thailand (1997-2000)	Pregnant women	Quinine + Clindamycin [5] Artesunate (AS)	Day 42 parasite failure, mean Hb, gametocyte carriage, adverse events

36	Gabon (2003-2004)	Children (3-12 yrs)	Quinine+clindamycin [6] Artesunate (AS2)+ clindamycin	Day 28 parasite failure, PCT, FCT, mean Hb, adverse events

Dosages: AQ: 25 mg/kg over 3 doses; CQ: 25 mg/kg over 3 days; SP: 500 mg/25 mg as single dose; AS: 12 mg/kg over 5 days; AS2: 2 mg/kg every 12 h for 6 doses; Dx: 2 mg/kg every 12 h for 6 doses; Tx: 4 mg/kg every 6 h for 7 days

[1] Quinine: 10 mg/kg every 12 h for 6 doses; clindamycin: 10 mg/kg every 12 h for 6 doses

[2] Quinine: 12 mg/kg every 12 h for 6 doses; clindamycin: 5 mg/kg every 12 h for 6 doses

[3] Quinine: 10 mg/kg every 8 h for 7 d; clindamycin 5 mg/kg every 6 h for 7 days

[4] Quinine: 8 mg/kg every 8 h for 3 days: clindamycin 5 mg/kg every 8 h for 3 days

[5] Quinine: 15 mg/kg every 12 h for 6 doses; clindamycin 7 mg/kg every 12 h for 6 doses

### Methodological quality in the included studies

Only one trial described how the allocation sequence was generated [36]. Three trials had adequate allocation concealment [36,38,39] and were therefore considered of high quality. In the remaining four trials, methods used for allocation concealment were unclear [34,35,37,40].

In only one trial, were participants, providers and outcome assessors adequately blinded using a double-blind and placebo-controlled design [39]. Two trials were open-label, suggesting that the patients and the providers were not blinded to the treatment assignment, but it was unclear in these trials whether the outcome assessors were also blinded [35,36]. In four trials, it was unclear if any attempt was made to blind the participants, providers or outcome assessors, as there was no mention of blinding in these trials [34,37,38,40].

Loss to follow up was below 10% in four trials [34,36,38,39]. The remaining three trials [35,37,40] excluded between 10 to 21% of study participants from analysis. Table 2 is a summary of the methodological quality assessment of the included studies.

**Table 2:** Risk of bias in the included trials

Study	Allocation sequence	Allocation concealment	Blinding	Number randomized	Loss to follow up N (%)
Kremsner 1988	Unclear	Unclear	Unclear	115	20 (17.4)

Kremsner 1994	Unclear	Unclear	Unclear	144	14 (9.7)

Metzger 1995	Unclear	Unclear	Open	120	12 (10)

Pukrittayakamee 2000	Unclear	Unclear	Unclear	204	43 (21)

Parola 2001	Unclear	Adequate	Double	115	7 (6.1)

McGready 2001	Unclear	Adequate	Unclear	131	2 (1.5)

Ramharter 2005	Adequate	Adequate	Open	100	5 (5)

### Efficacy of clindamycin plus quinine versus other anti-malarial drugs

**Figure 1:**
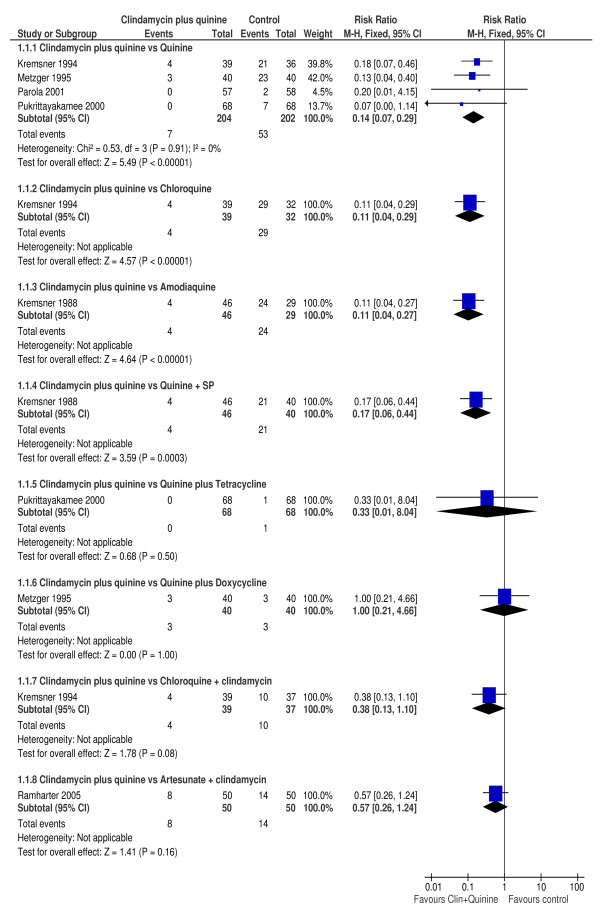
**Day 28 parasitological failure**.

There were nine comparisons between clindamycin plus quinine and the different control treatments. Figure 1 is a summary of the parasitological failure risks on day 28 between clindamycin plus quinine in comparison with the control treatments.

### Clindamycin plus quinine versus quinine

**Figure 2:**
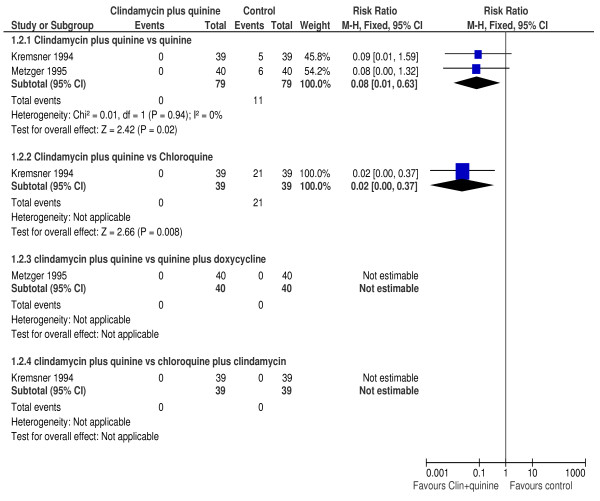
**Day 14 parasitological failure**.

In four trials [34,35,37,39], the proportion of participants experiencing parasitological failure on day 28 was significantly lower among those treated with clindamycin plus quinine compared with those treated with quinine (RR 0.14, 95% CI 0.07 to 0.29; 406 participants). PCR adjusted failure rates were significantly lower in those treated with clindamycin plus quinine compared to those treated with quinine alone (RR 0.13, 95%CI 0.04 to 0.40; 80 participants) in one trial [35]. In two trials [34,35], a significantly higher proportion of participants treated with quinine had parasitological failure on day 14 compared to those treated with clindamycin plus quinine (RR 0.08, 95% CI 0.01 to 0.63; 158 participants). Figure 2 is a summary of the participants with day 14 parasitological failure. There was no evidence of heterogeneity between these trials (I^2 ^= 0%). In one trial [37], the mean parasite clearance time was similar between participants treated with clindamycin plus quinine compared to those treated with quinine alone (WMD 2.0, 95% CI -5.61 to 9.61; 136 participants).

### Clindamycin plus quinine versus chloroquine

One trial [34], compared clindamycin plus quinine with chloroquine in 71 participants. The risk of parasitological failure by day 28 was significantly lower among those treated with clindamycin plus quinine compared to those treated using chloroquine (RR 0.11, 95% CI 0.04 to 0.29). Similarly, the risk day 14 parasitological failure was significantly lower among participants on the clindamycin plus quinine compared with those in the chloroquine arm (RR 0.02, 95%CI 0.0 to 0.37). The median parasite clearance time was significantly longer in the chloroquine arm compared to the clindamycin plus quinine arm: 5.5 vs 3 days, respectively.

### Clindamycin plus quinine versus amodiaquine

In one trial [40], clindamycin plus quinine was compared with amodiaquine in 75 participants. Compared to amodiaquine, the risk of parasitological failure by day 28 was significantly lower among those treated with clindamycin plus quinine (RR 0.11, 95% CI 0.04 to 0.27). The mean parasite clearance times were not statistically significantly different between the two regimes: 86.4 vs 82.7 h for clindamycin plus quinine vs amodiaquine, respectively.

### Clindamycin plus quinine versus artesunate

No participant failed treatment by day 28 in the single trial [38], which evaluated clindamycin plus quinine in comparison of with artesunate for the treatment of 131 pregnant women with uncomplicated falciparum malaria in Thailand. The parasite clearance time was significantly longer for participants treated with clindamycin plus quinine compared with those treated using artesunate (WMD 14.4 95%CI 14.03 to 14.77). However, a significantly higher proportion of participants developed gametocytes after treatment with clindamycin plus quinine compared with artesunate (RR 2.90, 95% CI 1.13 to 7.47). At the end of day 42 follow up, participants treated with clindamycin plus quinine also had a significantly lower mean haemoglobin compared to those treated with artesunate (WMD 0.66, 95% CI 0.18 to 1.14; 129 participants).

### Clindamycin plus quinine versus quinine plus sulphadoxine/pyrimethamine

In one trial [40], the risk of parasitological failure by day 28 was significantly lower among those treated with clindamycin plus quinine compared to those treated using quinine plus sulphadoxine/pyrimethamine (RR 0.17, 95% CI 0.06 to 0.44; 86 participants). The mean parasite clearance time was significantly longer with clindamycin plus quinine compared with quinine plus SP: 86.4 vs 78.2 h, *p *< 0.01.

### Clindamycin plus quinine versus quinine plus tetracycline

A similar risk of day 28 parasitological treatment failure was observed between those treated with clindamycin plus quinine compared to those treated using quinine plus tetracycline (RR 0.33, 95% CI 0.01 to 8.04; 136 participants) in one trial [37]. There was no difference in the mean parasite clearance time, 79 ± 20 vs. 77 ± 23 h, respectively. Similarly, the median FCT was comparable between the treatment arms, 8 (2-95) hours in the clindamycin plus quinine and 8 (3-36) hours in the quinine plus tetracycline arm, respectively.

### Clindamycin plus quinine versus quinine plus doxycycline

There was no difference in the risk of parasitological failure by day 28 when those treated with clindamycin plus quinine were compared to those treated using quinine plus doxycycline (RR 1.00, 95% CI 0.21 to 4.66; 80 participants). The mean parasite clearance time was similar between the two treatment regimes: 57.8 vs 52.8 h, respectively [35].

### Clindamycin plus quinine versus chloroquine plus clindamycin

The risk of parasitological failure by day 28 was not significantly different between those treated with clindamycin plus quinine compared to those treated with chloroquine plus clindamycin (RR 0.38, 95% CI 0.13 to 1.10; 76 participants) in one trial [34]. No participants failed therapy by day 14 on either arm. The median parasite clearance time was significantly shorter in the clindamycin plus quinine arm, 4 vs. 3 days, respectively.

### Clindamycin plus quinine versus artesunate plus clindamycin

Compared to artesunate plus clindamycin, comparable proportion of participants experienced parasitological failure on day 28 (RR 0.57, 95% CI 0.26 to 1.24), after PCR adjustment (RR 0.50, 95% CI 0.13 to 1.89) and similar proportions had gametocyte carriage (RR 2.50, 95% CI 0.51 to 12.29) after treatment with clindamycin plus quinine. Participants treated with clindamycin plus quinine had similar fever clearance time (WMD 9.0, 95% CI 0.78 to 17.22) and mean haemoglobin concentration (WMD 0.20, 95% CI -0.35 to 0.75) but significantly longer mean parasite clearance time (WMD 16.70, 95%CI 10.99 to 22.41) compared with those treated using artesunate plus clindamycin [36].

### Subgroup analysis

To investigate the effect of dosing regimens on parasitological failure by day 28, a sub-group analysis was performed for the comparisons between clindamycin plus quinine vs. quinine.

**Figure 3:**
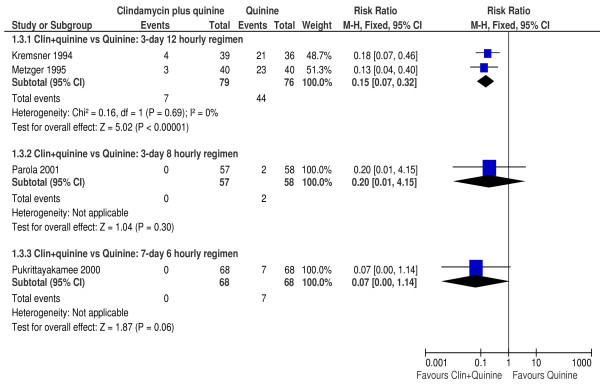
**Effect of treatment dosing on day 28 parasitological failure**.

In the two trials [34,35] where participants received the 12 hourly regime for 3 days, clindamycin plus quinine significantly reduced the risk of parasitological failure compared with quinine (RR 0.15, 95% CI 0.07 to 0.32; 155 participants). However, the risk of treatment failure was not significantly different between clindamycin plus quinine compared to quinine in the one trial [39] that evaluated the eight hourly treatment regime for 3 days (RR 0.20, 95% CI 0.01 to 4.15; 115 participants) or in the other trial [37] that evaluated the 6-hourly regimen for 7 days (RR 0.07, 95% CI 0.00 to 1.14; 136 participants). Figure 3 is a summary of the effect of dosing regimes on day 28 parasitological failure.

### Sensitivity analysis

**Figure 4:**
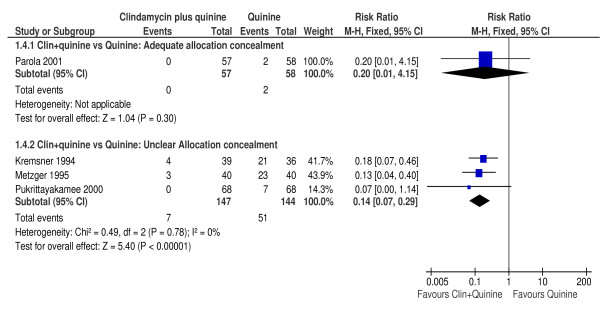
**Effect of allocation concealment on day 28 parasitological failure**.

Meta-analysis for the primary outcome was repeated separately using trials with adequate or unclear allocation concealment. This analysis was possible only for the comparison between clindamycin plus quinine compared with quinine monotherapy. There was no difference in the risk of treatment failure between clindamycin plus quinine compared with quinine by day 28 in the one trial [39] with adequate allocation concealment (RR 0.20, 95% CI 0.01 to 4.15; 115 participants). However, in the three trials [34,35,37] with unclear allocation concealment, treatment with clindamycin plus quinine significantly reduced the risk of treatment failure (RR 0.14, 95% CI 0.07 to 0.29; 291 participants). Figure 4 is a summary of the effect of allocation concealment on treatment efficacy.

### Adverse events

All the seven trials reported on adverse events. Four [36,38-40] of the seven trials described how adverse events were defined and assessed. Over all, five participants in two trials [39,40] experienced serious adverse events: watery diarrhoea due to *C. difficile *(two participants, treated with clindamycin plus quinine), severe haemolytic anaemia (one participant, treated with quinine), severe toxic rash (one participant, treated with quinine) and erythematous rash (one participant, treated with quinine plus SP). All the other adverse events were described as mild and transient.

## Discussion

This review summarizes the evidence from seven randomized controlled trials on the efficacy of clindamycin plus quinine in comparison with other anti-malarial drugs in the treatment of participants with uncomplicated falciparum malaria. The review aimed to determine whether clindamycin plus quinine is an effective and safe alternative for the ACT in the treatment of uncomplicated falciparum malaria. About 930 patients participated in the trials included in the review. Most of the included trials had relatively small sample sizes. A wide range of drugs were used in the comparisons and treatment regimens were often not similar.

This review had some limitations. Allocation concealment is an important measure of a trial's methodological quality. Only three trials adequately described their methods for allocation concealment. The risk of bias was therefore uncertain in four trials that did not describe these methods. Similarly, four trials did not clearly describe their blinding procedures and in three trials the rate of attrition was at least 10%, also suggesting a possible risk of bias. The reason for attrition was simply stated as loss to follow up--rendering it difficult to attribute to an effect of the interventions. Only two trials performed an intention-to-treat analysis [36,39]. Data was scarce for most comparisons, suggesting that this review may lack power to detect some differences. The primary outcome of this review is based on crude estimates of treatment failure. In malaria treatment trials, the true failure risk is best evaluated using the PCR technique, which distinguishes recrudescent (resistant) from new infections. In this review, only two trials used PCR to determine the treatment efficacy. Most trials had multiple arms and many treatment comparisons were derived from single trials. The available data was outdated and most of the studies were done with anti-malarial drugs that have long been phased out. Only one trial compared the efficacy of clindamycin plus quinine with an artesunate-based combination, albeit not a WHO-recommended ACT.

Within the mentioned limitations, the risk of parasitological failure by 28 days was significantly lower in participants treated with clindamycin plus quinine compared to quinine, amodiaquine, chloroquine, or quinine plus SP. A similar risk of treatment failure was noted between clindamycin plus quinine and quinine plus tetracycline, quinine plus doxycycline, artesunate plus clindamycin, and chloroquine plus clindamycin. The addition of clindamycin to quinine (compared to quinine monotherapy), improved treatment efficacy, shortened the duration of treatment and reduced the risk of treatment failure. In comparison to quinine alone, the combination consistently reduced the risk of treatment failure by 86%, with a 95% confidence interval ranging from 71 to 93%. Quinine is still considered highly effective in sub-Saharan Africa, but a decline in the sensitivity of quinine has been reported from several endemic areas suggesting that quinine resistance may already be present [41-44]. There are concerns that the emergence of quinine resistance may compromise the efficacy of this combination.

In all the trials reviewed, the combination appeared safe and serious adverse events were rare. However, the reporting of adverse event details was generally poor. The rapidity with which an anti-malarial drug relieves fever and clears parasites is an important consideration for ensuring treatment adherence. Clindamycin plus quinine was not consistently associated with shorter or longer fever and parasite clearance times.

The second edition of the WHO guidelines for treatment of malaria, recommends clindamycin plus quinine as the drug of choice for treating malaria infection in the first trimester of pregnancy and as a second-line drug for treatment failures, for malaria in the second and third trimesters of pregnancy, for travellers and for the oral phase of severe malaria treatment [7]. These guidelines recommend that the combination should be given for 7 days. This review suggests that in comparison with quinine monotherapy, a three-day course of clindamycin plus quinine (administered every 12 h) may be as effective as a 7 day course at reducing the risk of day 28 treatment failure. However, further study and direct comparison of dose regimens is required. The implementation of this combination may be limited by the high cost of clindamycin, the emerging evidence of quinine resistance and by the co-administration of the drugs (currently not co-formulated). Compared to artemisinin-based combination therapies, clindamycin plus quinine has limited effect on gametocytes, although this could be improved by adding primaquine to the therapy.

There was limited data for most comparisons, but this review suggests that clindamycin plus quinine is an effective treatment alternative for uncomplicated falciparum malaria that can safely be administered to both pregnant women and children. Sufficient evidence was found to indicate that clindamycin plus quinine is more effective at reducing the risk of parasitological failure by day 28 compared with quinine monotherapy. However, data is lacking on the efficacy of clindamycin plus quinine in comparison with artemisinin-based combinations for the treatment of uncomplicated malaria or even for the treatment of malaria in pregnancy. Adequately powered efficacy trials are urgently required to determine the efficacy of the three-day 12 hourly regimen of clindamycin plus quinine in comparison to artemisinin-based combinations in the treatment of uncomplicated malaria. Research is also required to establish the role of clindamycin plus quinine in the treatment of malaria in pregnancy. Better reporting of trial methods (especially, allocation concealment) would improve the interpretation of these future studies.

## Conflict of interest

The authors declare that they have no competing interests.

## Authors' contributions

COO developed the protocol, scanned the results of the literature search for potentially relevant trials, assessed potentially relevant trials for inclusion into the review, assessed the methodological quality of the included trials, independently extracted the data, entered the data into Review Manager, performed the statistical analysis, and drafted the manuscript. EAJ edited the protocol, assessed potentially relevant trials for inclusion into the review, assessed the methodological quality of the included trials and independently extracted the data. Both authors read and approved the final manuscript.
